# Retrieval-Augmented Generation for Medical Question Answering on a Heart Failure Dataset: Performance Analysis

**DOI:** 10.2196/84932

**Published:** 2026-02-26

**Authors:** Shiran Zhang, Evelyn Phan, Pedro Velmovitsky, Quynh Pham, Scott Sanner

**Affiliations:** 1 Department of Mechanical & Industrial Engineering, Faculty of Applied Science & Engineering, University of Toronto, 27 King's College Circle, Toronto, ON, M5S 1A1, Canada, 1 416-978-2011; 2 The Edward S. Rogers Sr. Department of Electrical and Computer Engineering, Faculty of Applied Science & Engineering, University of Toronto, Toronto, ON, Canada; 3 Centre for Digital Therapeutics, Toronto General Hospital Research Institute, University Health Network, Toronto, ON, Canada; 4 Institute of Health Policy, Management, and Evaluation, Dalla Lana School of Public Health, University of Toronto, Toronto, ON, Canada; 5 Telfer School of Management, University of Ottawa, Ottawa, ON, Canada; 6 School of Public Health Sciences, Faculty of Health, University of Waterloo, Waterloo, ON, Canada; 7 Department of Mechanical & Industrial Engineering, Faculty of Applied Science & Engineering, University of Toronto, Toronto, ON, Canada

**Keywords:** medical question-answering, retrieval-augmented generation, information retrieval, heart failure, healthcare technology, machine learning

## Abstract

**Background:**

The integration of retrieval-augmented generation (RAG) systems into the domain of medical question-answering (QA) presents a significant opportunity to enhance the effectiveness and accuracy of clinical support systems.

**Objective:**

This study aimed to explore the design choices within the RAG framework and the use of large language model (LLM) classifiers to optimize medical QA systems, enhancing response quality for patient and caregiver queries of varying risk levels.

**Methods:**

In total, we curated a dataset of 109 patient and caregiver questions related to heart failure (HF)—categorized into answerable (direct, fact-based queries), helpful deferral (general guidance or lifestyle advisory queries), and nonanswerable (out-of-scope or high-risk and medical intervention queries) types—along with relevant documents and a target answer for each question from the website *The Heart Hub*. Applying a system architecture leveraging RAG with a structured query taxonomy and robust classification mechanisms, this paper provided an empirical assessment for medical QA on a HF dataset and introduced a QA system pipeline design, providing a foundation for extended application across various medical fields. Specifically, we evaluated design choices in the initial retrieval stage of RAG and their impact on performance. We assessed final answer quality from the generation stage using popular passage scoring methods for QA, such as Recall-Oriented Understudy for Gisting Evaluation (ROUGE), BERTScore, and Intersection over Union score.

**Results:**

The pipeline first uses an LLM-based classifier, achieving 65% accuracy for answerable and helpful deferral queries and 100% accuracy for identifying nonanswerable queries. In information retrieval, the BioMedical Contrastive Pre-trained Transformers (MedCPT) cross encoder performed best as a dense retrieval method, delivering an average of 93% recall @ 7 through ranked relevance scores to obtain the top documents with recall @ k denoting recall computed over the top-k retrieved items. For further retrieving snippets from such documents, its average performance was 72.5% for sentence-level snippets and 83% for paragraph-level snippets. A second LLM-based classifier, used to refine the generated responses, resulted in an overall reduction in ROUGE-1 recall by 13% and Bidirectional Encoder Representations from Transformers (BERT) precision by 11%. However, Intersection over Union scores, or the overlap between “gold answers” and system answers, increased by 24%, demonstrating enhanced alignment with ground truth responses. This also indicates the system’s improved ability to generate concise and accurate medical responses.

**Conclusions:**

The implementation of a structured RAG framework paired with LLM classifiers for medical QA introduces a promising avenue for enhancing clinical decision support systems. By systematically analyzing the impact of query taxonomy, retrieval configurations, and response strategies, this approach clarifies the relative importance of each component within the medical RAG system using a HF dataset. Our findings provide actionable guidance on optimal design choices for maximizing retrieval and response accuracy; thus, informing the development of robust, scalable medical QA systems.

## Introduction

### Background

Medical question-answering (QA) systems, which can efficiently retrieve evidence-based medical information and deliver it to individuals during a dialogue appropriate to their knowledge level, have the potential to improve patient outcomes and enable better health self-management [1-4]. Such interactions have become more accessible with the recent introduction of generative artificial intelligence (GenAI) large language models (LLMs) as it integrates conversational dialogues that are not restricted by limited vocabulary and controlled inputs. LLMs possess advanced natural language understanding and conversational capabilities, enabling natural and engaging interactions while providing useful and relevant information. This is made possible through the transformer architecture, which is foundational for maintaining dialogue context and handling complex texts, such as the ones in the medical field [5].

However, LLMs also have limitations that might limit their use in real-world, high-risk scenarios, such as the potential of generating inaccurate responses (“hallucinations”). These hallucinations could, at best, spread misinformation and, at worst, lead to patient harm [1-3]. LLMs often lack domain-specific knowledge not included in their training data, which could result in hallucinations or outdated answers. Models like Google’s Med-PaLM [6], fine-tuned for health care, have been reported to include incorrect or inappropriate content, exhibit faulty reasoning processes, and occasionally contain biases in answers [7]. These inaccuracies, coupled with consumer feedback indicating lower helpfulness, highlight challenges in adaptability and specificity when addressing medical queries, especially in handling domain-specific questions. Other advanced open-source LLMs tailored for the biomedical domain, such as BioMistral [8] and Meditron [9], have sophisticated training on specialized corpora and adaptations for specific medical contexts but are still subject to issues including hallucinations, dynamic adaptation to new medical guidelines, and maintaining relevance across diverse medical specialties. This is primarily due to their initial designs that focus on large corpora and databases comprising a wide range of topics in the biomedical domain, such as PubMed Central, MEDLINE, and the Metathesaurus [10].

This knowledge gap can be addressed by retrieval-augmented generation (RAG), which improves performance by incorporating external knowledge (eg, evidence-based or peer-reviewed documents) and enhancing the system’s accuracy and relevance in medical contexts [7].

**Figure 1.**
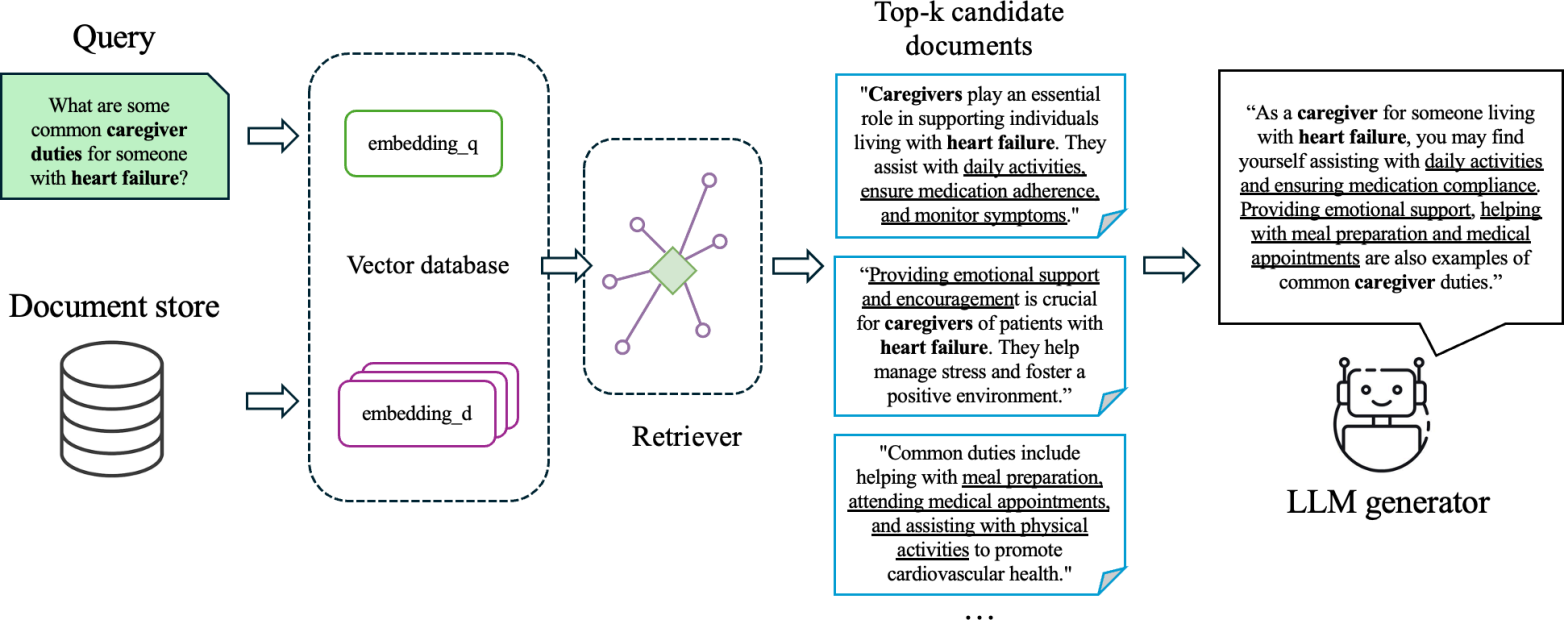
Overview of the retrieval-augmented generation (answer retrieval) pipeline. The system retrieves relevant documents from a vector database based on a user query and provides them as context to a large language model to generate a response. LLM: large language model.

RAG is a highly popular method for performing QA tasks by using an information retrieval (IR) system to retrieve a small set of candidate documents that are then provided as context for an LLM that is prompted to produce an answer to the question (if it can be answered from the candidate documents). In Figure 1, for the example user query “What are some common caregiver duties for someone with heart failure?” and “What are some common caregiver duties for someone with heart failure?” the system first retrieves the most relevant documents from a document store. These top candidate documents contain key information, which is then passed as context to an LLM generator, which uses the provided text to construct a single, coherent answer. IR models, such as BioBERT [11], MiniLM [12], and TAS-B [13] are commonly used in these stages, leveraging dense retrieval methods to improve the semantic understanding of complex medical queries.

In addition to reducing hallucinations, RAG enhances the verifiability of LLM outputs by grounding answers in retrieved references and minimizes the presence of outdated information [14]. The sensitive nature of evidence-based health information in medical QA applications requires robust systems that are safeguarded against hallucinations and maintain tight dialogue control while enabling natural, dynamic user interactions and the delivery of accurate information.

Earlier medical QA methods relied on structured formats, like translating medical questions into SPARQL queries to retrieve information from structured medical databases [10]. This approach enabled handling multiple query types, including questions with more than one focus or expected answer type. However, their system faced challenges in scalability and required additional mechanisms to address more complex queries, such as “why” or “when” questions. Other approaches implemented natural language processing techniques, such as syntactic chunkers, to extract noun phrases in query generation and answer extraction [15]. However, such methods are highly dependent on the quality of the chunker’s training data. This reliance was found to limit their ability to capture medical terminology, which hinders the retrieval of critical information in a medical context.

Building on earlier retrieval-based techniques, subsequent research integrated deep learning models to improve both the answer extraction and response generation stages. Early hybrid systems combined traditional sparse retrieval with advanced neural networks. For example, Yang et al [16] used a Bidirectional Encoder Representations from Transformers (BERT)–based “reader” to extract answers from documents initially retrieved by BM25 more accurately. A more foundational breakthrough, however, occurred in the retrieval stage with the shift to dense methods. The introduction of dense passage retrieval by Karpukhin et al [17] substantially improved the relevance of retrieved documents by embedding questions and passages in a shared vector space. This architecture of a dense retriever paired with a generative model became the foundation for modern RAG systems, later validated by benchmarks from Xiong et al [18] on a vanilla RAG system that highlighted improvements in both retrieval accuracy and final response quality.

In contrast to previous approaches, this paper introduces an enhanced RAG system featuring LLM-based classifiers designed to answer a variety of query types and optimize each stage in the pipeline. While it can be used in and scaled to other domains, the system was designed with a focus on health care, namely for the high-risk context of answering heart failure (HF) queries as part of an integrated chat feature in a digital HF management module. Our system categorizes and answers queries based on their nature and topic sensitivity levels. This enables broad query coverage and results in a comprehensive system of QA for the clinical domain of HF, and our methods can easily be extended through other similar domains in health care. Through systematic testing and evaluation, we identify the optimal system configuration and design choices for each stage, leading to a more effective RAG framework.

### Goal of Study and Paper Structure

Our research leverages LLMs within an RAG framework to design and evaluate an LLM-based medical QA system with a specific focus on HF. This condition was chosen as it is one of the “fastest growing cardiovascular conditions in the world” [19], with over 750,000 Canadians living with HF and 100,000 new cases being diagnosed annually [20]. Digital interventions have been shown to improve outcomes in patients with HF and lead to better self-care. Medly (Centre for Digital Therapeutics) is an evidence-based digital therapeutic that enables University Health Network (UHN) patients in Canada to monitor weight, heart rate, blood pressure, and symptoms, as well as output alerts and actionable self-care feedback [21]. Patients can also talk to nurses during working hours through a Messenger feature, asking questions about HF and their course of treatment. However, they are not able to promptly communicate with their care team after work hours. In this manner, an LLM-enabled medical QA system for HF can be included as part of Medly Messenger to improve patient engagement and self-management of their conditions, allowing patients to receive guidance and resourcing anytime. The medical QA system described in this work serves as a first step toward having an after-hours chatbot for Medly, although its principles and architecture can be reused in domains other than HF.

To create our medical QA system, we first introduce a structured taxonomy of question types in this domain. Questions were devised by the study authors after consultation with nurses from UHN and medical students from the University of Toronto, ensuring the system is tuned to practical, real-world medical scenarios. Ground-truth answers were obtained from the HF-centric website *The Heart Hub* by the Ted Rogers Center for Heart Research [22], an evidence-based resource typically used by nurses to educate patients. With these resources, we developed a system architecture that leverages LLMs with specific instructions for each task—from question classification to IR and answer generation—which is adaptable and applicable to a wide range of complex medical QA scenarios.

The rest of the paper is structured as follows. First, the methods are presented, including a description of the query taxonomy, the dataset used, and the system architecture with the individual LLM classifiers. The results of the evaluation on a set of predefined questions are also presented. Finally, we discuss these results and the optimal design choices found during the evaluation and conclude the paper.

## Methods

### Dataset and Query Classification

The scope of the queries in the dataset was defined by the content of The Heart Hub, an educational resource developed by the Ted Rogers Center for Heart Research to support patients living with HF and their caregivers [22]. Having consulted clinicians and medical researchers from UHN, *The Heart Hub* was identified as an appropriate resource reflecting the range of topics relevant in HF education and built in partnership with people with lived experience in HF. As a result, the dataset includes queries addressing HF directly, as well as related cardiovascular topics, related conditions, treatments, and symptom management guidance covered within the source material and frequently discussed in the context of HF.

Based on the frequency of topic appearance, the medical relevance and urgency of the information, and common misconceptions about heart health, we generated a dataset of 109 potential patient and caregiver queries. To compile such queries, all webpages on *The Heart Hub* were first extracted using a web crawler and stored as individual documents, which served as contextual input to GPT-3.5-Turbo (OpenAI) [23]. A few sample raw documents can be found in Multimedia Appendix 1. The model was prompted to generate factual query-response pairs reflecting common informational needs and concerns of patients and caregivers regarding HF and related topics covered in the corpus. This ensures a comprehensive representation of patient and caregiver concerns.

In this study, we selected GPT-3.5-Turbo, an advanced state-of-the-art language model developed by OpenAI [23], among various LLMs for its unique advantages in processing complex medical data. While there are newer and larger models like GPT-4 (OpenAI), GPT-3.5-Turbo not only excels in accurately understanding and generating contextually relevant text, but it is also more cost-effective due to its open-source nature and requires less computing power [23,24].

Given the critical importance of accuracy and reliability in automated QA systems within the medical domain, we implemented measures to mitigate associated risks, such as misdiagnosis and misinformation. To achieve this, ground truth answers were instructed to be strictly extractive from the source text and limited to a maximum of 5 sentences to ensure concision while fully addressing each query. These sentences either directly answer the query or provide helpful information to enhance user understanding. The ground truths served as a standard for evaluating the system’s performance, enabling the measurement of its adherence to the source material. To verify the quality of the dataset, members of the research team manually reviewed the generated query-response pairs to ensure relevance and alignment with the source material. Ground truth answers were considered sufficient if they directly addressed the query or provided contextually relevant information without introducing content beyond the source material.

To further address potential inaccuracies and the critical need for reliable information, we developed a taxonomy to categorize queries based on their associated risk. The development of this taxonomy was guided by discussions with medical researchers at UHN, who provided sample patient and caregiver queries that helped inform the identification of varying levels of urgency. Based on these considerations, queries were organized into 3 distinct categories—answerable, nonanswerable, and helpful deferral questions. More information on these 3 categories of query types is provided in Table 1.

**Table 1.** Taxonomy of medical queries categorized by risk level. Definitions and examples illustrate how the system distinguishes between direct factual answers, helpful deferrals, and out-of-scope or high-risk queries.

Query type	Query type definition	Example query	Ground truth for example query
Answerable	Fact-based queries that require direct information responses such as medical conditions, patient and caregiver experiences, or available resources.	What tests are used to diagnose heart failure?	Detecting heart failure can be difficult because symptoms may be confused with other conditions. Blood tests, electrocardiogram (ECG), echocardiogram (ECHO), and other imaging tests like chest X-rays are commonly used to diagnose heart failure.
Helpful deferral	Queries that are related to personal health management, lifestyle choices, and safety and precautions.	What lifestyle changes can I make to manage heart failure?	Personalized professional help from healthcare providers like dietitians, pharmacists, or your primary care physician will be more helpful for your individual needs. However, in general, a healthy lifestyle is essential. For someone with heart failure, this means paying extra attention to diet, getting regular exercise, avoiding alcohol, and taking care of your mental well-being.
Nonanswerable	Queries that either fall outside of the corpus’s scope or pertain to situations requiring immediate medical intervention, accurate diagnosis, or specific treatment recommendations.	What specific medications can I take that are currently recommended for heart failure?	Sorry, I don’t have enough information to answer your question. Please feel free to try another question. If you have any concerns or need detailed advice, please consult a professional such as a healthcare provider, dietitian, pharmacist, or your primary care physician.

Query type labels were assigned using a multistage process. First, few-shot prompting was applied using GPT-3.5-Turbo to generate initial labels for all queries based on the predefined taxonomy. These labels were then reviewed by members of the research team (EP and SZ). Queries identified as ambiguous or potentially misclassified were reviewed by both research team members, who agreed on the final label. While a third reviewer would have been consulted if disagreements remained, all queries were resolved by consensus.

This taxonomy establishes a foundational framework for determining the appropriate approach when answering queries, prioritizing patient well-being and promoting safe and effective interaction between automated systems and users. Each query in the dataset was systematically categorized according to the taxonomy. Of the 109 queries, 66 were classified as answerable, 17 as helpful-deferral, and 26 as nonanswerable. The full list of the 3 types of queries is listed in Multimedia Appendix 2.

**Figure 2.**
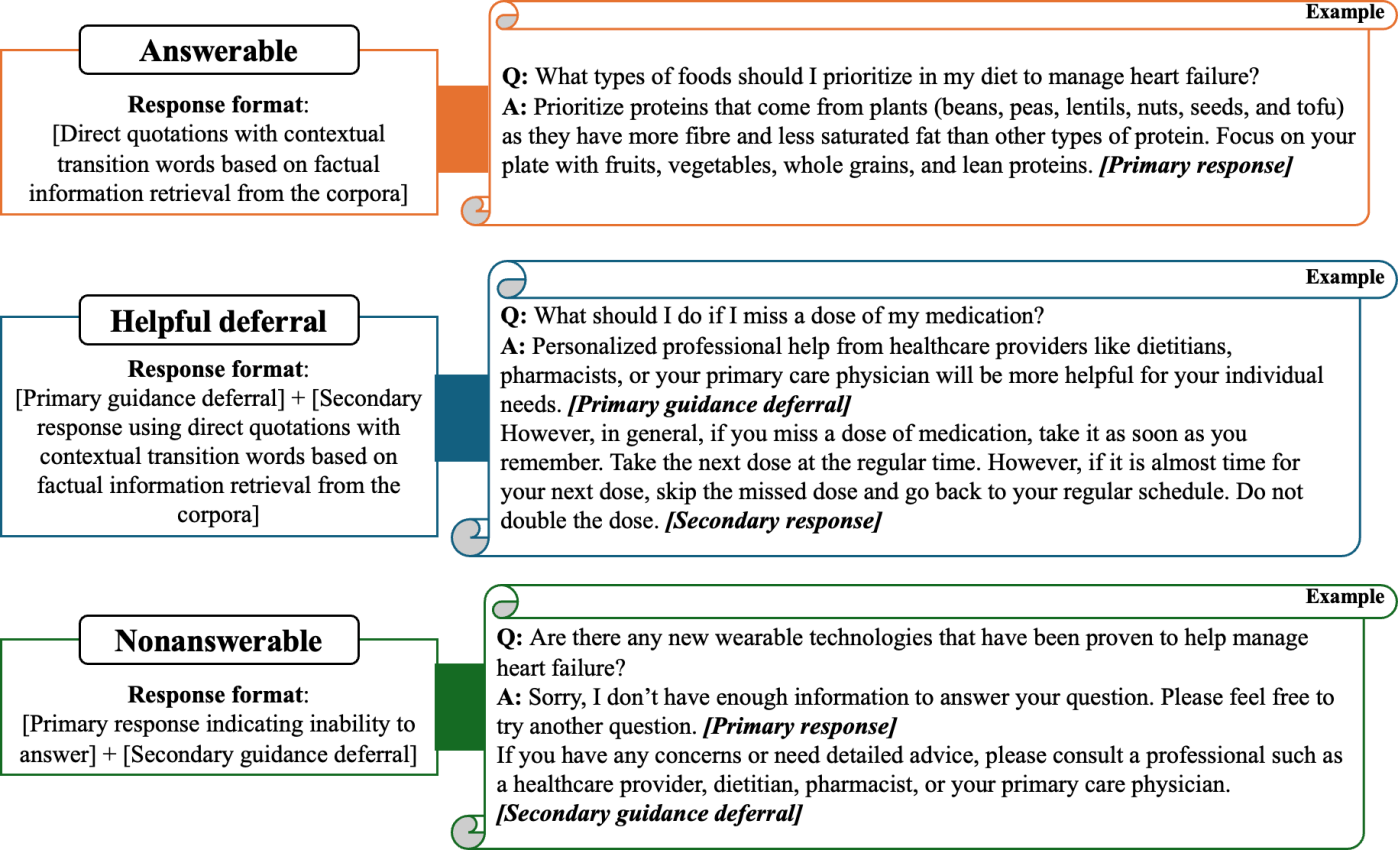
Response templates for the medical query taxonomy. These formats integrate the placement of professional guidance and cautionary statements to ensure user safety when providing information for sensitive or complex health queries.

In alignment with the established taxonomy, we designed a corresponding response format to address the varying risk levels associated with the queries, as detailed in Figure 2. For answerable queries, which present minimal risk, the response consists solely of direct extractive quotes from the corpus. For nonanswerable queries that fall outside of the corpus’ scope or involve high-risk situations, a predefined response is used. This response first indicates the system’s inability to provide an answer, then advises the user to seek professional medical assistance to address any remaining concerns. For helpful deferral queries, the response format includes direct quotes from the corpus, preceded by a cautionary statement recommending that users consult a health care professional for personalized advice. This precautionary statement is placed at the beginning of the response to ensure it is prominently read and understood, thereby prioritizing user safety and reducing potential liability for the system. This structured approach, informed by the query taxonomy, enhances the robustness of the system and supports safe and effective user interaction.

### System Architecture

**Figure 3.**
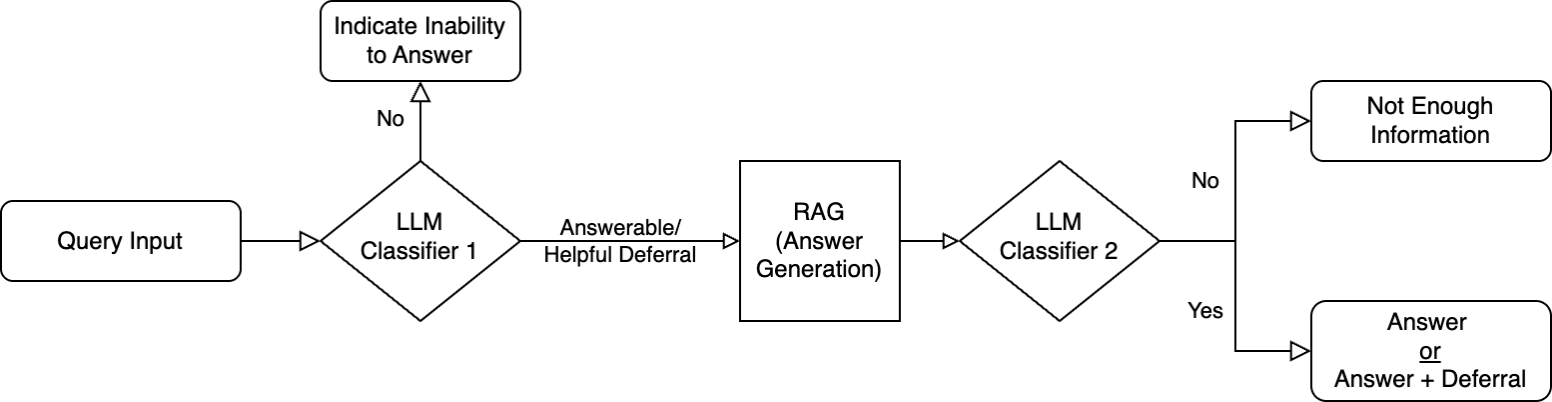
Proposed medical retrieval-augmented generation system pipeline. An initial large language model classifier categorizes input queries to determine answer feasibility. A secondary classifier then evaluates the answer generated through retrieval-augmented generation before delivering the final response. LLM: large language model; RAG: retrieval-augmented generation.

The proposed medical RAG QA system uses a pipelined approach, as illustrated in Figure 3. Initially, incoming queries undergo categorization to determine the appropriate response format. For queries categorized as answerable and helpful deferral, the system implements the RAG process. This involves retrieving relevant information from the corpus through the IR and snippet retrieval stages. The generated response then undergoes verification and refinement before being presented to the user. Further details regarding each stage of the pipeline are outlined in the subsequent sections. The full implementation of the proposed system pipeline, including source code and experimental configurations, is available in Multimedia Appendix 3, where a link to the GitHub repository is included.

### LLM Classifier 1

The initial stage of the pipeline uses GPT-3.5-Turbo to categorize queries into 1 of the 3 predefined taxonomy categories. In this phase, our objective is to mitigate the risk of providing inappropriate or harmful responses by preventing the system from answering high-risk, nonanswerable queries, thereby safeguarding user interactions. We evaluated both zero-shot and few-shot [25] prompting techniques with chain-of-thought prompting [26]. Zero-shot prompting autonomously generates responses without examples, while few-shot prompting enhances the model’s output by supplying it with specific examples, thus illustrating the desired response format and reasoning process to improve categorization accuracy. Our iterative refinements revealed that using 5 examples per category at this stage significantly enhances the effectiveness of the few-shot, chain-of-thought approach, optimizing the system’s ability to accurately categorize and respond to queries. The finalized prompt is presented in Multimedia Appendix 4. Additionally, the chain-of-thought technique facilitated the verification of the model’s reasoning process and ensured consistency in the output. To address the inherent inconsistency of LLMs, the prompt was designed to encourage conservative and cautious classifications, prioritizing patient safety and minimizing the risk of misinformation.

### RAG

#### IR

Following the initial categorization, the system proceeds to the retrieval stage, an essential component of the RAG pipeline, as showcased in Figure 1. This phase aims to identify and retrieve the most relevant documents by comparing the similarity between the embedded query and corpus documents [27]. Dense retrieval methods are designed to capture semantic and contextual relationships between queries and documents, leveraging vector representations that allow for more accurate matching compared with traditional keyword-based approaches [28]. This makes dense retrieval particularly effective in complex domains, such as medical information, where precise semantic understanding is critical. In contrast, sparse methods (like BM25 [29,30]) rely on exact keyword matching and are less capable of understanding deeper contextual relations. The design choices of the IR stage explored in this study include a range of dense retrieval models, namely paraphrase-MiniLM-L6-v2 [31], GPT-3 [25], BioMedical Contrastive Pre-trained Transformers (MedCPT) [32], BioBERT [11], BioMedBERT [33], and TAS-B [13].

Subsequent to document retrieval, the system refines the extracted information through snippet extraction. This process involves segmenting the relevant documents into smaller units, such as sentences, paragraphs, or sections as defined by text segments between header tags of the original web page, and using a retriever model to select segments that are the best match for answering the query [17]. By collecting high-quality snippets, the language model in the subsequent stage of the RAG system is better equipped to generate more precise and accurate responses. Similar to document retrieval, dense retrieval methods were used to capture semantic nuances within the snippets [28]. As such, the models tested in this stage include paraphrase-MiniLM-L6-v2 [31], GPT-3 [25], MedCPT cross encoder [32], BioBERT [11], BioMedBERT [33], and TAS-B [13]. Adjusting the length of snippets is crucial to gage the granularity at which these retrieval models can accurately identify and match sentences from the ground truths, thus enhancing response alignment accuracy. It is important to note that due to inconsistent HTML formatting on the *The Heart Hub* website, snippet lengths were restricted to sentences or paragraphs.

#### LLM Answer Generation

The final stage of RAG involves generating responses to user queries based on the relevant snippets retrieved in the preceding stage. To enhance response quality, we evaluated both zero-shot and few-shot chain-of-thought prompting techniques on the GPT-3.5-Turbo language model. The model was tasked with leveraging the information from the retrieved snippets to produce responses that are accurate, concise, and directly address the user’s query. This process requires selecting the most relevant sentences from the snippets and delivering answers that are purely extractive, all while adhering to the predefined answer formats. The finalized few-shot prompt used in the experiments is included in Multimedia Appendix 5.

### LLM Classifier 2

To mitigate the potential inconsistencies and inaccuracies inherent in LLMs [34], a second layer of verification was implemented using another LLM-based classifier. This classifier was designed to assess whether the generated response adequately addressed the user’s query. If the response is deemed relevant and suitable, it is refined using the prompt in Multimedia Appendix 6, and the most pertinent information that answers the question is extracted. This condenses the responses to align with the ideal length for clarity and precision, as GPT-3.5-Turbo sometimes generates overly lengthy responses. For most queries tested, this resulted in responses being condensed to only a few sentences. Conversely, if the response is deemed irrelevant or inaccurate, the system discards the generated answer and produces a nonanswerable query output, thus preventing the system from providing misleading or incorrect information. This multistage approach was developed to minimize misinformation and maximize the accuracy and quality of the responses.

### Evaluation

#### Recall-Oriented Understudy for Gisting Evaluation

To evaluate the performance of the generated responses, we implemented the Recall-Oriented Understudy for Gisting Evaluation (ROUGE) metrics, which are established tools for assessing the quality of automated text summarization and machine translation systems [35]. Specifically, the ROUGE-1 recall metric was used, which measures the proportion of unigrams (individual words) in the generated response that are also present in the ground truth [35]. It is calculated by dividing the number of exact unigram matches without stemming by the total number of unigrams in the ground truth. A higher ROUGE-1 recall score indicates a greater degree of overlap between the generated response and the ground truth, reflecting the system’s effectiveness in adhering to the source material.

#### BERTScore

BERT is a deep learning model developed by Google for natural language processing [36]. Unlike ROUGE, which focuses on surface-level exact word matching, BERT captures more sophisticated semantic meaning through contextual word embeddings. BERTScore is a metric designed to leverage the pretrained contextual embeddings from BERT to evaluate text quality [37]. It measures the semantic similarity between the generated text and a reference text by comparing the vector representations of words or phrases, known as word embeddings, in both texts. In particular, we used the BERT precision metric, which calculates the average similarity of the word embeddings in the generated text to the most similar embeddings in the reference text. Unlike ROUGE-1 that only captures surface-level unigram matches, BERT precision provides a deeper understanding of how closely the generated responses semantically align with the ground truth.

#### Intersection over Union

Intersection over Union (IoU) is a metric originally developed for evaluating object detection performance in computer vision, where it measures the extent of overlap between predicted and ground truth boxes in an image [38]. As demonstrated in the following equation, “*Ans*” refers to the predicted answer span, and “*GT*” is the ground truth span.


$$IntersectionoverUnion(IoU)=\frac{\left|Ans\cap GT\right|}{\left|Ans\cup GT\right|}=\frac{CommonconsecutivewordsbetweenAnsandGT}{{{Ans}_{length}+GT}_{length}-CommonconsecutivewordsbetweenAnsandGT}$$

**Figure 4.**
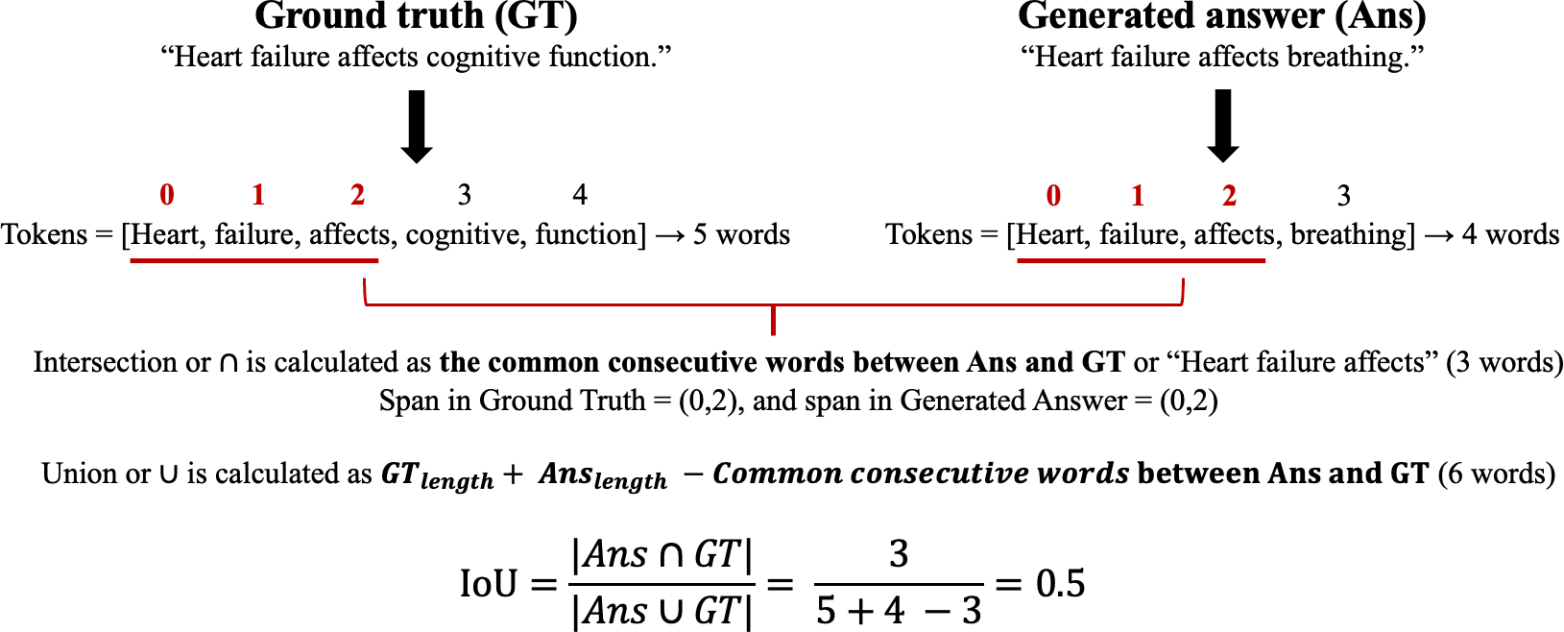
Example of Intersection over Union score calculation. The score identifies the ratio of shared consecutive words between the ground truth and the generated answer to quantify the degree of overlap. IoU: Intersection over Union.

When adapted for text evaluation, IoU quantifies the degree of overlap between generated responses and ground truth answers [37,39]. The numerator represents the number of elements common between the predicted answer and the ground truth, and the denominator is the total number of unique elements in both the predicted answer and the ground truth. In this case, IoU is computed by summing the length of all common substrings between the generated text and the ground truth, then dividing this value by the union of the generated answer and the ground truth, as illustrated by an example in Figure 4.

This approach allows IoU to determine how closely the generated response matches the ground truth while penalizing answers that are longer than necessary, which ROUGE and BERTScore do not address. As shown in previous works like NLQuADind, IoU score can serve as an evaluation metric for the purpose of assessing sequence similarities [39]. It effectively shows that while all scores, such as *F*_1_, ROUGE-1, ROUGE-2, and ROUGE-L, behave similarly in the higher values, the limitation of these metrics is that they overestimate the performance in the lower IoU values due to a higher chance of overlap between the bag of words, n-grams, or longer longest common subsequences in the prediction and target spans [39]. Using IoU, we can ensure generated answers are both extractive and concise, minimizing the risk of misinformation.

#### Recall for Retrieval

To evaluate the performance of the retrieval stage, we used standard recall to assess the effectiveness of each design choice. First, recall was measured by determining whether the correct document containing the ground truth sentences was retrieved, with experiments conducted using different retriever models to select the top 3, 5, and 7 documents. In the snippet retrieval stage, recall was used to quantify the proportion of ground truth sentences among the extracted snippets, with tests conducted on snippet sizes of 15, 20, and 30 sentences, as well as 3, 5, and 10 paragraphs. This analysis aimed to determine the most effective snippet length for maximizing relevant information while minimizing irrelevant content. These evaluations provided insights into the performance of each retrieval stage, allowing for the optimization of design choices and enhancing the system’s ability to deliver precise and relevant information in response to user queries.

#### Classifier Prompt-Based Evaluation

The performance of the classifiers was evaluated by using a prompt-based method to compare the outputs of the language models to the taxonomy labels for classifier 1 and the relevance of responses for classifier 2. Classifier 1 was evaluated by assessing whether the model’s categorization of each query matched the predefined dataset categories. This involved calculating the proportion of correctly classified queries within each category to gage the model’s effectiveness. Classifier 2 was evaluated on whether the model’s assessment of the generated response’s relevance aligned with the intended ground truth answer. A classification was considered accurate if the model correctly identified the presence or absence of relevant information, supported by a valid reasoning process. Through such evaluations, we were able to assess the effectiveness of each classifier in ensuring relevant responses and appropriate query handling.

### Ethical Considerations

The study did not involve any human participants, human participant interaction, or expert or professional involvement. No personally identifiable information was used, and all data were derived from the publicly accessible *The Heart Hub* website. All annotations, labeling, and analyses were performed by the coauthors and related team members. As such, no ethics board approval was required.

## Results

### Overview

Based on the pipeline flowchart as illustrated in Figure 1, a testing methodology was designed to validate the efficacy of our system across its various components, with each evaluated separately to ensure comprehensive assessment. For each of the 3 query types, we developed a testing strategy which ensures that responses are tailored to the nature of the query, thereby optimizing safety and relevance in patient interactions. As mentioned in previous sections, testing measures such as ROUGE, BERTScore, Recall @ K, and IoU scores were used.

### Evaluation Outcomes

Classifier 1 efficiently categorizes incoming queries into 3 types, setting the stage for a tailored response generation for user queries. As illustrated in the following contingency matrix (Table 2), the accuracy rate is 65% for answerable, 65% for helpful deferral, and 100% for nonanswerable queries, indicating robust classification for nonanswerable queries in particular.

**Table 2.** Classifier 1 accuracy contingency matrix. Accuracy rates demonstrate the system’s ability to correctly categorize incoming queries, with the highest success rate observed in identifying nonanswerable queries.

Actual class	Predicted answerable	Predicted helpful deferral	Predicted nonanswerable	Accuracy rate
Actual answerable	43	15	8	0.65
Actual helpful deferral	2	11	4	0.65
Actual nonanswerable	0	0	26	1.00

In the IR stage of the RAG components, the best dense retrieval models suitable for this task were the MedCPT cross-encoder and TAS-B. Using these models, the system scored a high score of up to 94% recall @ 7 for answerable questions and 92% for helpful deferral questions, as seen in Table 3, confirming the relevance of retrieved documents. Additionally, our experiments on snippet testing with an IR stage on MedCPT cross encoder further highlighted the effectiveness of paragraph-length answers over sentence-length, with paragraph recall @ 3 achieving up to 77% for directly answerable questions (Table 4).

**Table 3.** Results of information retrieval testing on sparse and dense retrieval models. High recall scores for the MedCPT cross-encoder and TAS-B highlight the effectiveness of dense retrieval in identifying relevant context.

Retrieval metric	BM25	Paraphrase-MiniLM-L6-v2	GPT3	MedCPT cross encoder	BioBERT	BioMedBERT	TAS-B
Answerable							
Recall @ 7	0.69	0.68	0.8	0.94	0.37	0.38	0.78
Recall @ 5	0.63	0.68	0.8	0.89	0.23	0.26	0.78
Recall @ 3	0.55	0.57	0.78	0.8	0.09	0.15	0.74
Helpful deferral							
Recall @ 7	0.56	0.8	0.84	0.92	0.44	0.48	0.92
Recall @ 5	0.52	0.8	0.84	0.92	0.16	0.36	0.92
Recall @ 3	0.52	0.68	0.8	0.92	0	0.32	0.76

**Table 4.** Results of snippet testing with information retrieval model of MedCPT cross encoder, k=5, and both sentence and paragraph-based snippet length, as indicated by their respective headers. These results demonstrate the impact of context size in IR, as paragraph-length snippets consistently achieve higher recall for both query types.

Retrieval metric	Whoosh TF-IDF	Paraphrase-MiniLM-L6-v2	GPT3	MedCPT cross encoder	BioBERT	BioMedBERT	TAS-B
Answerable (sentence-based)							
Recall @ 30	0.20	0.55	0.70	0.72	0.59	0.40	0.62
Recall @ 20	0.19	0.53	0.68	0.66	0.41	0.29	0.56
Recall @ 15	0.17	0.45	0.56	0.59	0.38	0.23	0.46
Helpful deferral (sentence-based)							
Recall @ 30	0.19	0.65	0.76	0.73	0.63	0.44	0.65
Recall @ 20	0.19	0.64	0.70	0.68	0.57	0.43	0.62
Recall @ 15	0.19	0.49	0.65	0.68	0.51	0.41	0.53
Answerable (paragraph-based)							
Recall @ 10	0.48	0.47	0.85	0.87	0.39	0.26	0.77
Recall @ 5	0.43	0.39	0.81	0.77	0.27	0.21	0.72
Recall @ 3	0.37	0.35	0.77	0.70	0.21	0.17	0.65
Helpful deferral (paragraph-based)							
Recall @ 10	0.47	0.65	0.82	0.79	0.35	0.47	0.82
Recall @ 5	0.41	0.59	0.65	0.65	0.29	0.29	0.68
Recall @ 3	0.29	0.53	0.59	0.53	0.24	0.29	0.68

In the final answer generation within the RAG system, we experimented with both zero- and few-shot prompting strategies to obtain a comprehensive evaluation of the system’s capability to handle complex medical queries with high accuracy and reliability. Moreover, as Table 5 illustrates, results gathered using zero-shot prompting highlighted a nuanced performance with and without the snippet extraction step. Notably, queries generally performed better without snippet extraction, with answerable questions achieving a ROUGE-1 recall of 75% compared with 63% with snippets. Few-shot prompting, on the other hand, was more conservative and context-specific, showing improved results across all categories. In particular, for helpful deferral questions, few-shot testing without snippets showed an average ROUGE-1 recall of 61%, whereas the zero-shot approach resulted in an average of 57% only.

**Table 5.** Zero-shot comparison testing of with or without snippet retrieval stage. The results demonstrate the system’s ability to obtain higher evaluation scores when processing full document context over isolated sections.

Evaluation condition	Rouge-1 recall	Bert precision	IoU score
Answerable
With Snippets	0.63	0.71	0.23
Without Snippets	0.75	0.76	0.28
Helpful deferral			
With Snippets	0.45	0.61	0.21
Without Snippets	0.57	0.69	0.25

To further examine the effectiveness of the system, we have conducted comparison testing to evaluate specific performances of quantifiable improvements achieved by classifier 2 modifications. By examining the detailed comparisons, we were able to assess the system’s adaptability and precision in managing diverse query complexities.

Following response generation, classifier 2 plays a critical role in ensuring relevance and accuracy by assessing if the answers generated meet the stringent criteria required for medical information. On average, classifier 2 showed strong accuracy in verifying against the dataset derived from the *The Heart Hub* website, achieving scores of 91% for answerable, 71% for helpful deferral, and 96% for nonanswerable questions. It is important to note here that, based on previous testing procedures which determined that performance without snippets surpassed that with snippets, we exclusively used the method of testing without snippets in the before-and-after assessments of classifier 2. Moreover, the impact of classifier 2 is evident in the before-and-after comparison, where improvements in the precision and interpretability of responses were noted.

**Figure 5.**
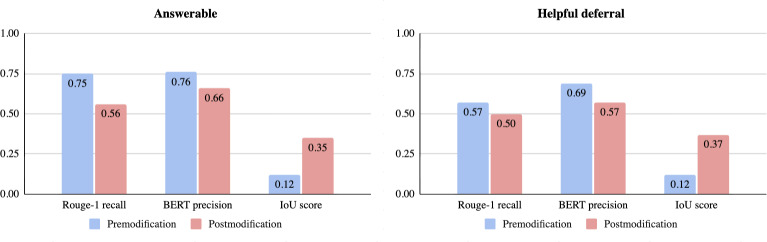
Premodification and postmodification results by classifier 2. The charts show a significant increase in Intersection over Union scores postmodification, indicating that responses become more concise and focused even as ROUGE-1 and BERT Precision scores decrease due to shorter output length. IoU: Intersection over Union.

As Figure 5 illustrates, the postmodification results demonstrated improved IoU scores, underscoring the classifier’s efficiency in refining responses. For instance, the IoU score for helpful deferral questions increased from 0.12 to 0.37, illustrating the classifier’s effectiveness in refining and validating system outputs. Admittedly, there is a slight drop in the other 2 metrics—shorter outputs tend to include fewer reference unigrams (lower ROUGE-1 recall) and may align less at the token level even when conveying the same information (lower BERT precision). However, the higher IoU score still suggests the revised responses are more targeted to the relevant context, despite being more concise. This is because classifier 2, whose prompt is included in Multimedia Appendix 6, assesses whether each generated output effectively answers the query with binary decisions (yes or no) after the refinement. Having the final judgment supported with logical reasoning as required by the output template, we can be sure that none of the important clinical details was deleted. As such, despite the dip in ROUGE-1 recall and BERT precision, the result of a higher IoU score shows improvement as it produces responses that prioritize accuracy and relevance while being shorter and easy for users to read and understand.

## Discussion

### Principal Results

This section evaluates the performance of the system’s components, including query classification, IR, and answer generation. This analysis provides valuable insights into optimizing both retrieval and classification stages to enhance the system’s ability to deliver precise, reliable, and safe information in response to HF medical queries. The feasibility of the system is reflected by the consistent end-to-end pipeline execution across all configurations and stable generation without any null or incomplete answers. Acceptability of successful outputs is assessed based on the performance at each stage of the pipeline, such as appropriate deferrals of queries, the alignment with ground truth evidence, and output conciseness.

### LLM Classifier 1 Failure Analysis

Despite the generally accurate query classifications, as indicated by the accuracy rates reflected in the main diagonal of the contingency matrix in Table 2, classifier 1 was designed to favor more conservative or safer classifications, represented by the upper right triangle of the matrix. Notably, for 93% of inaccurately categorized queries, the classifier exhibited this conservative tendency, highlighting its effectiveness in prioritizing safety. This focus on safety is particularly important in the medical domain, and considering the final goal is to implement a similar system as part of Medly Messenger. Prioritizing caution can help prevent the delivery of harmful advice or recommendations that could compromise patient health.

Several queries categorized under the helpful deferral category were inaccurately labeled as answerable. Queries that are advisory in nature and may not always require personalized advice based on patient history were placed in the helpful deferral category to be more conservative and ensure patients and their caregivers receive the best quality advice. However, because advisory and precautionary queries can occasionally be generalized, the LLM sometimes classified them as answerable. For example, the query “How can I manage my heart failure care effectively?” was classified in the helpful deferral category due to its potentially personalized nature but was misclassified as answerable, as general advice on HF could be provided. This misclassification highlights ambiguity in the classification system, potentially leading to responses that may not be adequately safe or appropriate for higher-risk topics.

In cases where the categorization was clearer and more straightforward, the LLM showed greater consistency in identifying the correct question category. This was particularly true for nonpersonal informational queries, such as those requesting general medical facts or treatment guidelines, where the distinction of an answerable query was clear. Similarly, the model performed reliably for most helpful deferral queries related to personal health management. These patterns suggest that the LLM excelled when the queries involved nonsensitive topics or when it was evident that personalized responses were required. However, several patterns of misclassification were observed:

- *Answerable queries misclassified as helpful deferral*: Queries involving potentially personal topics were occasionally classified as helpful deferral despite being general advisory queries. An example of one of these misclassified queries is, “What can I do if I notice an increase in my water weight?” This was the most common misclassification, accounting for 51.7% (15/29) of incorrectly classified queries.
- *Answerable queries misclassified as nonanswerable*: Some queries addressing topics requiring medical attention or risks of complications related to medications were categorized as nonanswerable. An example is, “What should I do if I experience dizziness or lightheadedness while taking ACE inhibitors or ARBs?” This type of misclassification constituted 27.6% (8/29) of the total misclassifications.
- *Helpful deferral queries misclassified as nonanswerable*: Queries related to medications were often misclassified as nonanswerable, likely due to the sensitivity of these topics. For instance, the query “Can I take ACE inhibitors or ARBs with or without food?” was misclassified in this manner. This issue had a prevalence similar to the last category, making up 13.8% (4/29) of incorrectly classified queries.

These misclassifications indicate the challenges the classifier faces, particularly in distinguishing between general advisory queries and those requiring more personalized or sensitive responses.

### IR Design Choices and Strategies

In the IR stage, it is evident that the MedCPT cross encoder demonstrated superior performance compared with other retriever models. This outcome is expected in comparison to sparse methods that rely on keyword matching. Indeed, sparse methods frequently struggle with accurate document retrieval due to frequent occurrences of the same keywords across different contexts, a common challenge in medical IR. In contrast, dense methods are more adept at understanding the meaning and context of queries, resulting in the effective retrieval of relevant documents.

MedCPT’s strong performance is potentially due to its specialized training for medical contexts, using 255 million query-article pairs from PubMed search logs [32]. This extensive training in medical literature enhances MedCPT’s ability to understand and retrieve pertinent information within the medical domain more effectively than other models. Additionally, the MedCPT cross-encoder implements a unique reranker that uses cross-attention computation between query and article tokens, providing greater accuracy compared with the dot product similarity used for the other models [28]. While other domain-specific retrievers like BioBERT, fine-tuned specifically for biomedical text mining [11], and BioMedBERT, which focuses on biomedical terminologies and concepts [33], are also trained on biomedical data, their specialized training may not align perfectly with the diverse types of queries and documents encountered in this context. This misalignment could contribute to these models’ relatively lower performance.

Similar to the document retrieval stage, the MedCPT cross-encoder demonstrated superior performance overall in snippet retrieval. The document retrieval task benefits from specialized, domain-specific models like MedCPT, which excels in understanding and retrieving contextually relevant documents. However, it is important to note that snippet retrieval can also benefit from general-purpose models, such as GPT-3 and TAS-B, for their broad text processing capabilities as presented in Table 4 [13,17].

As expected, recall for snippet retrieval improved with the number of sentences or paragraphs extracted. Some models exhibited reduced performance with paragraph snippets, likely due to fewer opportunities to select relevant snippets. Nevertheless, models that performed well with sentence snippets also demonstrated enhanced recall with paragraph snippets. This improvement is due to the fact that paragraphs often contain consecutive sentences, which provide additional context that may not be captured in individual sentence snippets. However, inconsistencies in the HTML structure of the website occasionally led to inaccuracies in paragraph snippet grouping, reducing their overall reliability. Therefore, for the remaining experiments, we opted for sentence snippet retrieval with 30 sentences, which was the most effective configuration for this task.

### Answer Generation Performance

For the initial answer generation stage, GPT-3.5-Turbo with zero-shot prompting achieved satisfactory results, particularly for answerable and nonanswerable queries. However, its performance significantly declined with few-shot prompting, likely due to the board variability in possible answers that few-shot examples struggled to capture comprehensively. As a result, the model often defaulted to not answering queries, as it could not effectively generalize from the examples provided. In contrast, when the same prompt was applied to GPT-4-Turbo, the model began to produce answers for most questions, demonstrating superior performance compared with GPT-3.5-Turbo. Specifically, GPT-4-Turbo generated responses that were not only more faithful to the context but also more concise. Additionally, we evaluated different system configurations, including a 2-stage approach, which involved IR followed directly by answer generation, and a 3-stage approach incorporating the snippet retrieval stage. The results revealed that the 2-stage system outperformed the 3-stage system, with average improvements of 12%, 6.5%, and 4.5% for ROUGE-1 recall, BERT precision, and IoU score, respectively, for answerable and helpful deferral queries. This superior performance of the 2-stage system is likely due to the high accuracy achieved in the IR stage, which diminished after the snippet retrieval stage, thereby reducing the likelihood of producing an accurate answer.

### LLM Classifier 2 Refinement

Finally, the system’s outputs were reassessed following the application of classifier 2, which refined the initial responses. The accuracy metric indicated that the classifier performed with high precision in identifying relevant responses from the initial answer-generation phase. Re-evaluating the system’s answers after refinement, as illustrated in Figure 3, reveals a notable increase in the IoU scores. This improvement suggests that classifier 2 effectively selected the most relevant sentences, thereby producing more concise and relevant responses. Despite a reduction in ROUGE-1 recall and BERT precision scores for answerable and helpful deferral queries, this reflects a strategic trade-off between minimizing misinformation and striving to match as many answers to the ground truth as possible. Prioritizing accuracy and safety, it is advantageous to use classifier 2 to verify the quality of responses, particularly given the inherent inconsistencies of LLMs and the critical need to prevent misinformation.

### Key Insights and Findings

In summary, this study presents several key insights into the performance of our medical QA system. Classifier 1 demonstrated solid accuracy in categorizing diverse medical queries, particularly by adopting a conservative approach that prioritizes safety, ensuring that sensitive queries are handled appropriately. While MedCPT proved to be the most effective model in both the IR and snippet retrieval stages, the 2-stage system, comprising only IR and answer generation, outperformed the setup that included snippet retrieval. This simplified approach, combined with zero-shot prompting for answer generation, produced the most accurate results by avoiding the challenges of generalizing from few-shot examples. Finally, incorporating classifier 2 at the end of the pipeline further enhanced response precision and concision, ensuring safer and more reliable answers, even at the expense of a slight reduction in lexical matching metrics. Overall, this approach highlights the critical balance between safety, accuracy, and quality in medical QA systems.

### Limitations and Future Work

Despite the promising performance of our system in the study, one noted limitation is the difficulty in handling medical terminology abbreviation and layman term recognition, such as “HF” for heart failure. Oftentimes, there is a disconnect between the medical language used by patients and medical practitioners, leading to confusing intent and mistakes [40]. Indeed, patients often use colloquial or more familiar medical terms, such as abbreviations and acronyms, rather than strict scientific or medical terminology, which also poses a challenge to our current system since the corpora used for responses primarily use formal medical language. This can lead to misunderstandings or incomplete information being relayed if the system fails to accurately interpret these commonly used variants. Since misinterpretation of medical abbreviations can lead to inaccuracies in information exchange, potentially affecting recommendations and patient understanding of their medical condition, we see a need in future work to integrate a comprehensive biomedical vocabulary dataset for abbreviation recognition and expansion to better understand and provide useful answers to user inquiries. Additional evaluation metrics, such as robustness [41], which measures the system’s resilience to handle disruptions like layman terms and adversarial attacks [3], should be included in the system.

**Figure 6.**
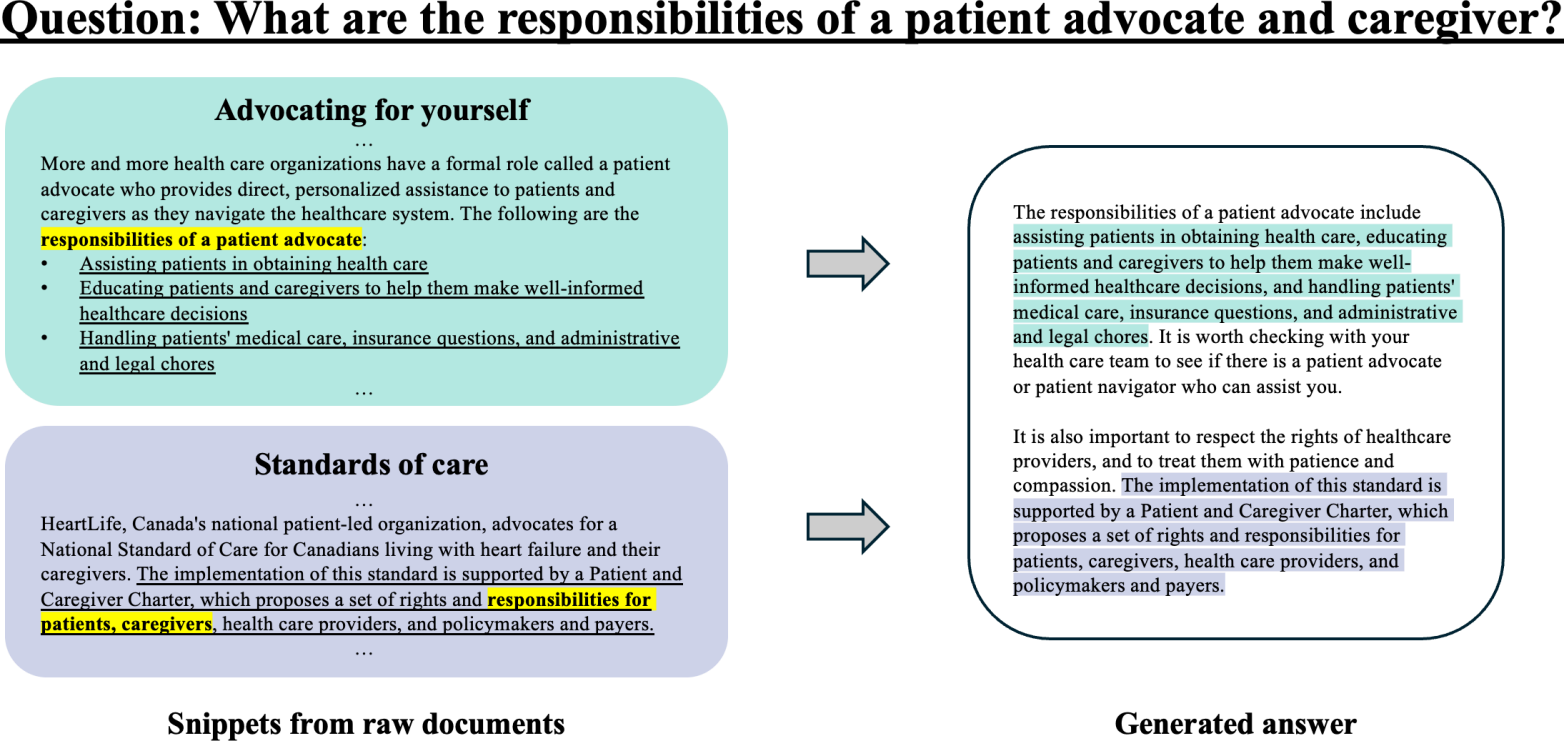
Example of a sample answer sourced from two distinct raw documents. This sample shows how the system can combine different sentences across documents to answer a query, which can lead to a lack of flow between ideas.

Another limitation is associated with the use of snippets for response generation. Specifically, crucial information may be omitted if snippets extracted from sources are not seamlessly integrated. This was evident in cases where consecutive sentences from different parts of a source resulted in missing contextual links, leading to incomplete or disjointed information being presented to the user. As Figure 6 illustrates, the challenge of maintaining coherence across separate information sources is highlighted. A potential solution is to refine the snippet selection and merging strategies to ensure that the extracted information fully captures the nuances and complete context of the query. This involves enhancing the LLM’s ability to understand and synthesize the thematic continuity between snippets, thereby improving the coherence and completeness of the responses.

Additionally, our system was designed to handle single-topic queries to ensure depth in responses. While not the focus of our study, handling multitopic inquiries simultaneously might be helpful in medical management for better efficiency and patient care. For future improvements, we seek to explore a strategy that processes multiple query components separately and then synthesizes the responses into a cohesive answer, ensuring comprehensive and contextually appropriate information is delivered.

The results and insights presented here can be used in the future to inform an implementation of an instance of the system within Medly for further evaluation. This study is limited by the methodological text similarity and overlap performance evaluation without any clinical testing. However, the balancing act between safety and quality should be tested against real queries of patients with HF and their perceptions of interacting with the chatbot collected to inform additional testing and refinement. In other words, the proposed medical HF QA system showed promise in creating a relatively safe and accurate prototype for answering HF queries. Next steps include the validation and further refinement of this prototype through co-design with patients with HF using the proposed architecture.

However, it is important to note that using LLMs in the medical domain presents ethical, legal, and safety concerns, which are still very much open-ended questions within health care organizations [3,42,43]. Issues, such as data privacy and the potential consequences of LLM responses, demand rigorous scrutiny and the implementation of stringent controls to prevent misuse and ensure the safety and fairness of applications [44]. In particular, preventive measures should be taken for jailbreak LLM prompts to ensure the system is not used to elicit incorrect or unsafe answers [45,46]. To avoid such attacks and misuse, countermeasures such as restricting question frequency limits and preprocessing user questions before classifying should be implemented. The patient co-design process can aid in the definition of security and privacy as well through the understanding of the optimal safety parameters for the system according to the end users. These parameters will be the gold standard against which clinical implementations should be evaluated against.

### Conclusions

This study investigated various design choices within the RAG framework to optimize medical QA systems. We examined the integration of classifiers into the RAG pipeline to address queries of varying risk levels and enhance the precision of answers generated by standard RAG. Our research encompassed a detailed evaluation of different retriever models and demonstrated that incorporating LLM-based classifiers significantly improves system performance. These classifiers contribute to generating more accurate and succinct responses by refining the system’s approach to query handling. While our approach can be generalizable to several domains, we examined its implementation for HF as a proof-of-concept chatbot to improve the Medly digital therapeutic. The results are promising and indicate that such a medical QA system applied to HF could be used as part of Medly to allow patients to have support anytime and anywhere.

Enhancing the accuracy and relevance of responses not only improves the overall user experience but also mitigates the risk of misinformation. This improvement can lead to better patient outcomes by providing reliable and timely information and support. Such advancements are particularly valuable in alleviating patient anxiety, especially during after-hours when access to medical professionals may be limited. By ensuring that patients receive accurate and reassuring information when needed most, this approach promises to enhance the overall effectiveness and reliability of automated medical QA systems.

## Supplementary material

10.2196/84932 Multimedia Appendix 1 Sample raw documents from The Heart Hub.

10.2196/84932 Multimedia Appendix 2 Complete queries, labels, and ground truths.

10.2196/84932 Multimedia Appendix 3 GitHub repository of source code.

10.2196/84932 Multimedia Appendix 4 Prompt used for Classifier 1.

10.2196/84932 Multimedia Appendix 5 Prompt used for answer generation.

10.2196/84932 Multimedia Appendix 6 Prompt used for Classifier 2.

## Abbreviations

BERT: Bidirectional Encoder Representations from Transformers
GenAI: generative artificial intelligence
HF: heart failure
IoU: Intersection over Union
IR: information retrieval
LLM: large language model
MedCPT: BioMedical Contrastive Pre-trained Transformers
QA: question-answering
RAG: retrieval-augmented generation
ROUGE: Recall-Oriented Understudy for Gisting Evaluation
UHN: University Health Network
