# Supplementary material for: Retrieval-Augmented Generation for Medical Question Answering on a Heart Failure Dataset: Performance Analysis
Source: JMIR Form Res. 2026 Feb 26;10:e84932. doi: 10.2196/84932 (PMC12945362; doi:10.2196/84932)
Supplement: Multimedia Appendix 1 [file formative-v10-e84932-s001.docx]

***Advance Care Planning.txt***

Most patients with heart failure will unfortunately die from the disease.

When approaching the end of one’s life, having an idea of what to expect and having a plan in place can relieve a lot of anxiety.

It is important to speak to your family or loved ones about your wishes now to help ease their distress if they must make difficult decisions in the future.

Advance care planning involves taking steps to ensure that your wishes guide your treatment and care if you become incapable and/or unable to communicate these preferences in the future.

There are many resources available to help you with advance care planning. Speak to your doctor for more information. Advance Care Planning

Heart failure is a serious illness. Most people living with heart failure will experience a gradual worsening and will eventually die from the disease. Although doctors may be able to give you a rough idea of your prognosis, it is impossible to predict how long a patient with heart failure will live.

Knowing what to expect and having a plan in place will help to relieve anxiety for you and your loved ones.

Advance care planning helps prepare you and your family for decisions they might have to make in the future.

What is advance care planning?

Advance care planning is the process of thinking about and communicating your wishes, values, and beliefs. You consider what is important and makes your life meaningful and take steps to ensure that your wishes guide your treatment and care if you become incapable and/or unable to communicate these preferences in the future.

During this process, a capable person may express:

The values and preferences that should guide future decisions about their care in the event they are incapable of making treatment decisions.

Who they trust to act on their expressed wishes and make health care decisions in their best interests if they are not capable. This person (or people) is indicated in a Power of Attorney for Personal Care.

Advance care planning includes having conversations with close family and friends about your values and beliefs. It might include discussions about specific medical procedures as well – particularly as you become more unwell. Advance care planning is also about considering the experiences and people that you want around you when you receive care.

Key decisions can include:

Identifying your substitute decision-maker (SDM). Your SDM is the person a healthcare provider would consult if you became too sick or unwell to make decisions for your health.

How to help your SDM learn about you, your values, and what matters to you so that they are best informed if they are required to make a medical decision for you.

Speaking to your healthcare team about your future and preferences, including your feelings about interventions such as CPR.

Did you know that everyone in Ontario has an automatic SDM? If you wish to change the person who would speak for you, you can. You can appoint a Power of Attorney for Personal Care.

Learn more about automatic SDM’s.

Visit the Advance Care Planning Ontario website for more information on how to do so

Living with Advanced Heart Failure

Caring for someone with Advanced Heart Failure?

You aren’t alone.

10% of the nearly 750,000 Canadians living with heart failure have advanced heart failure. Download our new guide, Living with Advanced Heart Failure: Coping with Symptoms and Uncertainty, for tips on symptom management, self-care, and planning for the future.

Why is advance care planning important?

There are several reasons people find it helpful to prepare themselves and their SDM through advance care planning:

It decreases a lot of stress and worry by identifying your SDM(s) before a health crisis and allowing you to have control over who speaks for you.

It helps you and your SDM(s) to be more aware and knowledgeable about any of your health conditions and what to expect in the future.

It decreases distress and worry for your SDM, as they will be aware of their role, be prepared, and have guidance from you in the event they have to make decisions.

Remember, your SDM(s) do not make decisions for you unless you are not mentally capable of making decisions yourself. Thinking about this now allows you and your SDM(s) feel prepared and informed.

How do I document my advance care plan?

After you’ve talked with your SDM(s), your loved ones, and even your health care team, you can record your wishes however you want – in writing, through a video or audio recording, or by simply telling your SDM.

Your wishes are then acted upon by your SDM if they are required to make decisions. They will interpret your wishes in the specific situation and, along with the health care team, help to make decisions that respect your pre-determined wishes, values, and goals. This discussion should be revisited over time as your health, priorities, and wishes change.

Advance care planning resources

In Ontario, there is a workbook that helps guide your advance care planning with information and a set of questions to help you think, prepare, and communicate your thoughts, values, and wishes.

This workbook is publicly available from the Advance Care Planning Canada website.

Other resources include:

UHN’s Advance Care Planning

Health Care at Home

Advance Care Planning Ontario

SpeakUp Ontario

Hospice Palliative Care Ontario

Hospice Toronto

Caregiver Supports

***Diet.txt***

A healthy diet includes:

A variety of fruits and vegetables. Fruits and vegetables have important nutrients. They should make up half of your plate at lunch and dinner and be your primary snack choice.

Whole grains and whole grain products life whole grain bread, oats, corn, barley, farro, oatmeal, brown and wild rice, and quinoa.

Healthy protein sources such as legumes, nuts, fish, seafood, low fat dairy, and lean and unprocessed meat and poultry.

Prioritize proteins that come from plants (beans, peas, lentils, nuts, seeds, and tofu) as they have more fibre and less saturated fat than other types of protein.

Liquid non-tropical vegetable oils

Foods prepared with little or no salt

Water. Replace sugary drinks with water as your drink of choice.

Limited processed foods

Limited added sugars

No alcohol (or very limited, if at all).

Find Heart Healthy Recipes on UHN’s website.Canada’s Food Guide

Canada’s Food Guide is an eating plan created by Health Canada to help people in Canada make healthy food choices. It is available in different languages. Top tips include:

1. Focus on your plate.

Health Canada recommends a plate loaded with fruits, vegetables, whole grains, and lean proteins. These foods have been shown to reduce the risk of cardiovascular disease, including risk factors such as high blood pressure and elevated blood cholesterol.

Dairy is included as a protein. To reduce your saturated fat intake, choose low fat milk, cheese, and yogurt.

2. Eat more plant-based foods.

Choose beans, lentils, soy, and nuts more often. The goal to improve heart health is to reduce saturated fat, which comes mostly from animal-based foods such as beef, chicken, and dairy products.

3. Think “outside of the box.”

Choose more whole foods and fewer ultra-processed foods that are loaded with sugar, sodium, and saturated fat.

Shop the outside aisles at the grocery store, avoiding inner aisles where the processed foods are located.

4. Drink smart.

Choose water instead of sugar-sweetened beverages, which make up the main source of total sugars in the Canadian diet. Excess sugar is linked to obesity and heart disease. Make water your drink of choice!

5. Be mindful when eating.

Cook more often

Eat with others

Reflect on your eating habits

Enjoy your food

Be aware of food marketing, which can be misleading.

Canada’s Food Guide

Healthy Heart Portion Guide

The following guide will help you integrate foods that are low in saturated and trans-fat, high in fibre, and low in sodium into your diet in appropriate portions. Note that the amount of food you need depends on your age, gender, body size, and activity level.

For more information, speak to your health care provider about referring you to a dietitian.

Portion Size versus Serving Size

According to the National Institutes of Health, portion size and serving size are defined as follows:

Portion size is the amount of a specific food that you choose to eat at one time and is completely under your control. Be aware that many foods that come as a single portion contain multiple servings.

Serving size is the amount listed on a food package’s Nutrition Facts Label. The nutrition values on the label are for one serving size, as suggested by the food manufacturer. The Nutrition Facts Label and ingredients list on food packages can help you make informed choices about your diet.

Listed below are serving sizes for various food groups.

Fruits and Vegetables

Fruits and vegetables are an excellent source of vitamins and minerals.

Eat 7-10 servings of fruits and vegetables per day.

Choose dark green and orange vegetables and orange fruit more often.

Eat at least one dark green and one orange vegetable each day.

For more fibre, use fresh fruit and vegetables in place of juices.

Choose (example of 1 serving):

125ml (½ cup) fresh or frozen vegetables

250ml (1 cup) salad

1 piece of fresh fruit (the size of a tennis ball)

½ cup mixed fruit

125ml (½ cup) unsweetened fruit

Choose less often:

Buttered, creamed, or deep-fried vegetables

Brine-cured or pickled vegetables like sauerkraut

Unsweetened fruit juice

V8 juice

Canned fruits in heavy syrup

Coconut and coconut milk

Dairy and Alternatives

Milk products are an excellent source of calcium and vitamin D.

Eat 2-3 servings per day.

Try fortified, unsweetened plant-based beverages if you do not drink milk.

Choose (example of 1 serving):

250 ml (1 cup) skim or 1% milk

250 ml (1 cup) calcium-fortified unsweetened plant-based beverage

¼ cup skim milk powder

175 g (3/4 cup) fat-free yogurt

Choose less often:

Whole milk, 2% milk, goat milk, regular cream, light cream, sour cream, and whipping cream

Evaporated whole milk and condensed milk

Yogurt with a M.F. content greater than 2%

Regular ice-cream

Non-dairy substitutes made with coconut oil or hydrogenated oil, such as coconut milk or cream.

Fats and Oils

Fats are a valuable source of essential fatty acids and vitamins.

Choose 6-9 servings each day.

Limit the amount of saturated and trans-fat in your diet.

Unsaturated fats are ‘healthy fats.’ Examples of unsaturated fats include olive, canola, and avocado oil.

Many commercial and processed foods contain hidden fat. Choose these foods less often.

Choose (example of 1 serving):

5 ml (1 tsp) of unsaturated fats like canola, olive, and avocado oils

5 ml (1 tsp) margarine that is soft tub, non-hydrogenated, low in saturated fat OR 10 ml (2 tsp) light margarine

15 ml (1 tbsp) salad dressing made with recommended oils OR 30 ml (2 tbsp) calorie reduced salad dressings

2 tsp (10 ml) nut butter such as peanut butter or almond butter

Raw, dry roasted, or in-the-shell nuts (for example: 4 walnut halves, 8 almonds)

1/6 medium avocado

7.5 ml (1/2 tbsp) regular mayonnaise and mayonnaise type dressings OR 15 ml (1 tbsp.) light regular mayonnaise and mayonnaise type dressings

Choose less often:

Butter, hard margarine, lard, suet, hydrogenated oils, palm oil, coconut oil

Salad dressing containing cream cheese and/or bacon

Peanut butter made with hydrogenated oil

Nuts that are roasted in hydrogenated oils, salted, and/or chocolate covered.

Grain Products

Grain products are a source of carbohydrates and dietary fibre.

Eat 6-8 servings per day.

Breads can be high in sodium. Please read the Nutritional Facts table to ensure it is a low sodium choice.

Make at least half of your grain products whole grain each day.

Eat a variety of whole grains such as barley, brown rice, oats, and whole grain breads.

Choose (example of 1 serving):

1 slice of bread, preferably whole grain

½ whole-wheat pita

¼ whole-wheat bagel

½ chapatti or roti

½ matza

½ tortilla

½ hamburger or kaiser bun, or English muffin

250mL (1 cup) homemade soups made with beans, lentils, barley, and vegetables

2-4 whole-wheat, low-fat crackers (check the serving size)

125 mL (1/2 cup) starchy vegetables: corn, sweet potato, yam, cassava

1 small or ½ medium potato

125ml (1/2 cup) cooked brown rice, whole-wheat pasta, macaroni, noodles, kasha, barley, bulgur, quinoa

For cereals, check the Nutrition Facts Label and choose items with more than 15% daily value of fibre and less than 5% daily values of sodium:

80ml (1/3 cup) Bran Buds with Psyllium

190ml (3/4 cup) cooked oatmeal

175ml (2/3 cup) Shredded Wheat

125 mL (1/2 cup) Kellogg’s All Bran

Choose less often:

Salted or high-fat crackers, egg bread, cheese rolls, croissants

Commercial cakes and cookies, store-bought muffins, doughnuts, Danishes, waffles, pancakes

Potato chips, corn chips

Canned soups and dehydrated soup mixes

Frozen French fries

Rice and pasta convenience products (such as Kraft Dinner), egg pasta, fried noodles.

Meat and Alternatives

Protein foods, including meat and plant-based proteins, are an important part of healthy eating.

Eat 2-3 servings per day.

One serving of meat, fish, or chicken is about 3 ounces (90 g) cooked.

Cheese is high in sodium. Read the Nutritional Facts Label of cheese products before you buy them.

Have plant-based proteins such as beans, lentils, chickpeas, and split peas often.

Choose (example of 1 serving):

Fresh, frozen, and canned low-sodium fish packed in water

90 g poultry. Choose lean ground chicken or turkey when possible.

90 g lean cuts of beef, veal, lamb, pork, rabbit, or venison. Choose extra-lean ground beef when possible.

50 g of 15% M.F. cheese, low sodium

2 eggs OR 4 egg whites OR 125 ml (1/2 cup) liquid egg whites

150 mg (3/4 cup) firm tofu

175 ml (3/4 cup) cooked lentils, soybeans, chickpeas, navy beans, kidney beans

Choose less often:

Canned fish soaked in oil

Battered, fried, creamed, smoked, salted, or pickled fish, caviar, and squid

Duck, goose, poultry skin, basted poultry

Luncheon meat, back bacon, ham, sausages, wieners

Organ meats, spareribs

Processed cheese, full-fat cheese, cheese spreads.

Portion Sizes

Food portions are increasing. Larger portions might mean that you are eating more and gaining weight. Here are some ways you can eat and serve smaller portions.

At home:

Plate the suggested serving size for each person (children, teenagers, and adults) and put away the extra food for another meal

If you are still hungry, have extra salad or vegetables instead of extra dairy, grains, or meat

Pack up leftovers for lunch the next day

When eating out:

Skip or share the appetizers

Split the main dish with your companion

Put half of your plate in a to-go container for the next day’s lunch or dinner before you begin eating

Order a mini dessert or share a dessert

Avoid refills of sweetened beverages such as soft drinks, iced tea, and lemonade OR stick to water

When ordering takeout:

Order less

Add a homemade salad to the meal

Snacks:

Measure out the amount according to the serving size instead of eating straight from the bag or box.

Avoiding buying snacks such as cookies, chips, ice cream, store-bought muffins, and cake

Have healthy snacks, such as fruit, in plain sight

Groceries:

Buying in bulk may be budget friendly, but is not serving size friendly

Use small reusable containers/bags to repackage your snacks according to the serving size

Re-package bulk purchases into small portions before freezing them.

It is easy to overeat when you are not paying attention. Don’t eat while watching tv, reading or on your computer

Using a food diary for a few days can help you pay closer attention to what, how much, and how often you are eating.
