# Supplementary material for: Retrieval-Augmented Generation for Medical Question Answering on a Heart Failure Dataset: Performance Analysis
Source: JMIR Form Res. 2026 Feb 26;10:e84932. doi: 10.2196/84932 (PMC12945362; doi:10.2196/84932)
Supplement: Multimedia Appendix 3 [file formative-v10-e84932-s003.docx]

We have made our dataset and code publicly available at:

<https://github.com/D3Mlab/medical-qa>
