# Supplementary material for: Retrieval-Augmented Generation for Medical Question Answering on a Heart Failure Dataset: Performance Analysis
Source: JMIR Form Res. 2026 Feb 26;10:e84932. doi: 10.2196/84932 (PMC12945362; doi:10.2196/84932)
Supplement: Multimedia Appendix 5 [file formative-v10-e84932-s005.docx]

**Zero-shot Prompt Used for Answer Generation**

Given a question and context, answer the question by directly quoting up to 5 sentences from the given context. Only use enough sentences to answer the question.

If there is not enough context to answer the question and/or the question is classified as unanswerable, say the following two sentences: Sorry, I don’t have enough information to answer your question. Please feel free to try another question.

If the question is classified as helpful deferral, start your answer with: Personalized professional help from healthcare providers like dietitians, pharmacists, or your primary care physician will be more helpful for your individual needs. However, in general,

If the context you are extracting from contains a personal story, explicitly say whose story it is.

Question: {{query}}

Classification: {{classification}}

Context: {{context}}

**Few-shot Prompt Used for Answer Generation**

Given a question and context, answer the question by directly quoting up to 5 sentences from the given context. Only use enough sentences to answer the question.

If there is not enough context to answer the question and/or the question is classified as unanswerable, say the following two sentences: Sorry, I don’t have enough information to answer your question. Please feel free to try another question.

If the question is classified as helpful deferral, start your answer with: Personalized professional help from healthcare providers like dietitians, pharmacists, or your primary care physician will be more helpful for your individual needs. However, in general,

If the context you are extracting from contains a personal story, explicity say whose story it is.

Examples:

Question: What are the main functions of the atria and ventricles in the heart?

Classification: answerable

Context: The atria are responsible for collecting blood, while the ventricles are responsible for pumping the blood to the lungs or the body. In normal situations, the atria and ventricles work together. The atria contract and send blood to the ventricles, which in turn contract and send blood to the lungs and the body. While all the chambers in the heart are important, the main pumping chamber to circulate the blood to the body is the left ventricle. This electrical activity usually starts in the upper chambers of the heart (the atria) and is communicated to the bottom chambers (the ventricles), telling them to contract. Valves separate the atria, ventricles, and arteries. The electrical signal passes through the atria (causing them to contract) to the atrioventricular (AV) node, another specialized group of cells in the middle of the heart between the atria and the ventricles. The heart is divided into the right side and left side and composed of four chambers: two right and left top chambers (atria) and two right and left bottom chambers (ventricles). It is divided into the right side and left side and composed of four chambers: two right and left top chambers (atria) and two right and left bottom chambers (ventricles). Your Heart Function The heart circulates blood throughout the body. Valves separate the atria, ventricles, and arteries.What is Heart Failure? On the right side, the tricuspid valve separates the right atrium from the right ventricle. Once the electrical signal passes through the AV node, it travels through more specialized tissue (called the His-Purkinje system) and then divides into the “bundle branches,” which transmit the electrical signal to the ventricles, causing them to contract. Valves There are four valves in the heart whose function is to ensure one-way blood flow. The oxygenated blood is then returned to the left atrium and emptied into the left ventricle, which pumps the oxygenated blood to the body.

Answer: 'The atria are responsible for collecting blood, while the ventricles are responsible for pumping the blood to the lungs or the body.'

Question: I have heart failure, is it safe for me to have sex?

Classification: helpful deferral

Context: But if you are living with heart failure sexual activity – like all your activities – may need a bit of planning and careful consideration. You are not alone in thinking about sex and heart failure. Here are some ways to start the discussion: “I read somewhere that my heart failure can get in the way of having sex.” “I want to be intimate with my partner but I’m scared about what will happen to my heart.” “My partner is worried about us having sex and that it’s not safe for my heart.” “Is it safe to use Viagra if I’m having trouble with getting or keeping an erection?” In fact about 2 out of every 3 people living with heart failure experience some issue with sex and intimacy. Having heart failure does not mean that you cannot exercise. Sexual activity is not dangerous to your heart. You may need to gradually build up to this level of activity.Exercise and Heart Failure Exercising regularly helps people with heart failure live longer improves quality of life and decreases the need for heart failure related hospital admissions. Do not have sex if you are unwell have chest pain or are very short of breath. Common Sexual Problems Related to Heart Conditions Your heart condition and your medications may affect sexual desire and relationships in a number of ways. Is it safe for me to exercise? Heart Failure Becomes a Big Issue From 1995 to 2003 my biggest issues were still arrhythmias but now I was going in and out of heart failure. If you can walk at a reasonable speed on level ground or climb a set of 20 stairs then sexual activity is unlikely to affect your heart. Basically I have had heart failure all of my life. My Advice to Others with Heart Failure My best advice is to listen to your body and get to know when something’s wrong. In fact studies show numerous benefits of exercise in people with heart failure. If you have any concerns about your health, please consult a healthcare provider.

Answer: If you have any concerns about your health, please consult a healthcare provider.'Sexual activity is not dangerous to your heart. But if you are living with heart failure, sexual activity – like all your activities – may need a bit of planning and careful consideration.'

Question: {{query}}

Classification: {{classification}}

Context: {{context}}

Answer:
