# Supplementary material for: Retrieval-Augmented Generation for Medical Question Answering on a Heart Failure Dataset: Performance Analysis
Source: JMIR Form Res. 2026 Feb 26;10:e84932. doi: 10.2196/84932 (PMC12945362; doi:10.2196/84932)
Supplement: Multimedia Appendix 6 [file formative-v10-e84932-s006.docx]

**Prompt Used for Classifier 2**

Given a question and an answer, determine whether or not the answer fully answers the question.

If it answers the question, output the most important sentences that answer the question to form a final concise answer.

Only use the sentences in the form they are given.

If the sentence "Personalized professional help from healthcare providers like dietitians, pharmacists, or your primary care physician will be more helpful for your individual needs." is in the answer, include it in the final answer.

Output: If the question was fully answered: "yes"

If the question was not answered: "no"

Also output the final answer and the accompanying reasoning for the answer.

Only provide one output per question.

Examples:

Question: What treatment should I follow for my heart condition?

Answer: When caring for someone with advanced heart failure, it is important to understand what guides heart failure medication choices. Your health care team will measure your response to treatment, and it is crucial to be proactive in advocating for the best care. Most people with heart failure will need to continue medications long-term, many for the rest of their lives, and learning about self-care is essential in managing the condition. If you have any more concerns, please consult a healthcare provider.

Output:

Final Answer: N/A

Question Answered?: no

Reasoning: The answer speaks about medication in a general sense but the question is inquiring about the user specifically, so it is not answering the question.

Question: How do I troubleshoot issues with my home blood pressure monitor?

Answer: To troubleshoot issues with your home blood pressure monitor, it is recommended that you monitor your blood pressure once a week and take those readings to clinic appointments. If your blood pressure is consistently high, you may need medications to control it. Blood pressure can be controlled with one or more medications, and it can be difficult to predict how long you will need to be on them. Sometimes, high blood pressure is related to specific cancer treatment and may return to normal after treatment, but sometimes medication may be needed long-term. Monitoring your blood pressure at home is crucial for managing your health, and it can help your healthcare team determine the best course of action for your treatment. If you have any more concerns, please consult a healthcare provider.

Output:

Final Answer: N/A

Question Answered?: no

Reasoning: The answer speaks about why it is important to monitor blood pressure, but mentions nothing about how to specifically troubleshoot issues with the monitor, so it is not answering the question.

Question: How is atrial fibrillation diagnosed?

Answer: Atrial fibrillation is typically diagnosed with an ECG. It may also be detected with a heart monitor that is worn for an extended period of time. These tests may be ordered after an individual has a stroke to determine if atrial fibrillation has caused the stroke. If you have any more concerns, please consult a healthcare provider.

Output:

Final Answer: Atrial fibrillation is typically diagnosed with an ECG. It may also be detected with a heart monitor that is worn for an extended period of time. These tests may be ordered after an individual has a stroke to determine if atrial fibrillation has caused the stroke. If you have any more concerns, please consult a healthcare provider.

Question Answered?: yes

Reasoning: The answer successfully identifies that atrial fibrillation is diagnosed with an ECG, therefore answering the question.

Question: What are the two types of transthyretin amyloidosis?

Answer: There are two types of transthyretin (ATTR) amyloidosis: wild-type ATTR and mutant ATTR. Wild-type ATTR can create amyloid deposits over several years and is more common in people over the age of 70 years. Amyloid deposits form primarily in the heart, veins and arteries, and soft tissues. In mutant type ATTR, people are born with an abnormal transthyretin gene leading to more rapid amyloid deposition. The heart and nerves are the most common sites for amyloid deposits in mutant ATTR. If you have any more concerns, please consult a healthcare provider.

Output:

Final Answer: There are two types of transthyretin (ATTR) amyloidosis: wild-type ATTR and mutant ATTR. Wild-type ATTR can create amyloid deposits over several years and is more common in people over the age of 70 years. In mutant type ATTR, people are born with an abnormal transthyretin gene leading to more rapid amyloid deposition.

Question Answered?: yes

Reasoning: The answer successfully lists and describes both types of transthyretin amyloidosis, identifying them as wild-type and mutant, thus answering the question.

Question: {{question}}

Answer: {{answer}}

Output:
